# Editorial: Unravelling the role of the oral microbiome in cancer

**DOI:** 10.3389/froh.2025.1606144

**Published:** 2025-04-29

**Authors:** Julia S. Bruno, Ghanyah Al-Qadami, Divya Gopinath, Alexa M. G. A. Laheij

**Affiliations:** ^1^ Molecular Oncology Center, Hospital Sírio-Libanês, São Paulo, Brazil; ^2^ Health and Biosecurity Research Unit, CSIRO, Adelaide, SA, Australia; ^3^ Basic Medical and Dental Sciences Department, College of Dentistry, Ajman University, Ajman, United Arab Emirates; ^4^ Centre of Medical and Bio-Allied Health Sciences Research, Ajman University, Ajman, United Arab Emirates; ^5^ Department of Oral Medicine, Academic Centre for Dentistry Amsterdam, University of Amsterdam and VU University Amsterdam, Amsterdam, Netherlands; ^6^ Department of Oral and Maxillofacial Surgery, Amsterdam UMC, VU University, Amsterdam, Netherlands

**Keywords:** oral cancer, microbiota, biomarker, oncology, oral health

**Editorial on the Research Topic**
Unravelling the role of the oral microbiome in cancer

Oral squamous cell carcinomas are a group of head and neck cancers. Their global incidence is increasing, while prevalence varies by region. The primary risk factors for oral cancer remain tobacco use and alcohol consumption, although the role of human papillomavirus is increasingly recognised (1). However, the cause of cancer is multifactorial, and the development of a tumour is a multi-step process. Advancements in research have brought attention to the role of the oral microbiota in tumour development. Recently, polymicrobial communities have been recognised as an emerging hallmark and enabling characteristic of cancer. Microorganisms may be involved in cancer development, malignant progression and response to therapy. They are involved in tumour-promoting inflammation and genomic instability (2).

Beyond its role in cancer, the oral microbiota has been studied for its association with malignant progression and response to cancer therapy and for its link to cancer-related toxicities and life-threatening clinical outcomes. This editorial explores different aspects of the role of the oral microbiota in cancer, including its role in oral cancer development (Liang et al.), its diagnostic potential (van Dijk et al.), its effects on cancer-related (oral) toxicities (Zecha et al.) and the advanced model systems to better understand its role in health and disease (Gopinath et al.).

In oral microbiome research, there is an ongoing debate about the suitability of animal models as proxies for the human microbiome, given the inherent differences between human and rodent microbial communities. A comprehensive review by Gopinath et al. highlighted that, despite their differences from humans, rodents remain a valuable model due to their suitability for close monitoring of cancer development, cost-effectiveness, *ex vivo* analysis of tissue-specific toxicities and practical research timelines. Furthermore, animal models help to reduce the variability typically encountered in clinical settings while enabling precise control of environmental conditions and stress factors, providing deeper insights into the role of the microbiota in cancer development and progression (Gopinath et al.).

To better understand lifestyle risk factors such as smoking, animal models can provide valuable insights. The toxic components of tobacco lead to tissue alterations and may impact the microbial composition. In the study by Liang et al., healthy control and oral cancer mice were chronically exposed to tobacco smoke. The results showed that, in the first four weeks, there was no significant change in microbial diversity. However, over a longer period (16 weeks), microbial diversity decreased, with an increase in Firmicutes and *Proteobacteria*, and a decrease in Bacteroidota phyla (Liang et al.). This study highlights how chronic tobacco use leads to dysbiosis in the oral cavity of mice and as such contributes to cancer development. It is worth mentioning that the development of a human oral microbiota-associated mouse model by transplanting human saliva into germ-free mice, which achieves a high degree of donor similarity, is a key advance for future studies in this field (3) (Gopinath et al.).

The role of the oral microbiota in oral squamous cell carcinoma (OSCC) has been recognised, although it remains unclear whether specific microorganisms or microbial signatures are directly linked to the disease. A scoping review by van Dijk et al. analysed 30 studies with 1,677 subjects comparing microbial diversity between OSCC patients and healthy controls and found that *Fusobacteria* were significantly more abundant in OSCC patients, while *Actinobacteria* were more prevalent in healthy controls. *Firmicutes* were abundant in both groups, while *Spirochaetes* and *Bacteroidetes* showed mixed abundance patterns. Among specific species, *Fusobacterium nucleatum*, *Porphyromonas endodontalis*, and *Prevotella intermedia* were more abundant in OSCC patients, whereas *Veillonella parvula* showed varying prevalence across studies. These findings suggest that the oral microbiota could be used as a diagnostic tool for OSCC, but further research is needed to determine how best to apply this knowledge in clinical settings (van Dijk et al.).

Oral toxicities are common during and after cancer treatment, and they significantly impact patient quality of life. The type of treatment directly influences the toxicities experienced. In head and neck cancer, patients in middle-to-late clinical stages typically undergo radiochemotherapy, which increases the risk of oral mucositis, acute and chronic hyposalivation, radiation-related caries, and osteoradionecrosis. For haematologic malignancies, haematopoietic cell transplantation involves a complex treatment regimen, including chemotherapy, immunosuppressants, and, in some cases, total body irradiation. Many of these complications have been linked to changes in the oral microbiota, either as a secondary consequence or as a potential causative factor. An updated five-phase model of the pathophysiology of oral mucositis supports the role of microbiota alterations as a contributing factor (4). In line with previous studies, the study by Zecha et al. showed that oral mucositis in chemotherapy patients is associated with a lower Shannon diversity index, along with a decrease in *Prevotella*, *Fusobacterium*, *Selenomonas*, *Actinomyces*, and *Leptotrichia*. Furthermore, patients who developed neutropenic fever had a significantly lower diversity of oral microbiota at the onset of fever (Zecha et al.).

Early diagnosis of oral cancer is crucial to improve treatment outcomes, survival rates, and patient quality of life. The oral microbiota offers a promising, non-invasive, and cost-effective screening tool and a potential therapeutic target, particularly in high-risk individuals. This Research Topic brings together a collection of original articles and reviews that showcase the latest advances in research methods and expand our current understanding of oral microbiota dynamics in cancer patients.
